# Crossing the barrier: nanomedicine as a frontier therapy for neuropathic Gaucher disease type 3

**DOI:** 10.1097/MS9.0000000000004527

**Published:** 2025-12-09

**Authors:** Minahil Saif, Aeyza Nasir, Sabeen Shafique, Mahnoor Fatima, Muhammad Talha

**Affiliations:** a Department of Medicine, King Edward Medical University, Lahore, Pakistan; b Department of Medicine, Shaheed Ziaur Rahman Medical College, Rajshahi Medical University, Bogura, Bangladesh

*To the Editor,*

Gaucher disease type 3, a form of sphingolipidosis, is a rare lysosomal storage disorder caused by mutations in the GBA1 gene, resulting in the deficiency of the glucocerebrosidase enzyme. This leads to abnormal buildup of glucocerebroside within macrophages of multiple organs. Clinically, patients may present at any age with pancytopenia, hepatosplenomegaly, bone crisis, and seizures. Unlike other forms, neurological involvement makes its management challenging^[1]^. Current therapies like enzyme replacement therapies and substrate reduction therapies are facing a major setback due to their inability to cross the blood–brain barrier (BBB), leaving the neurodegenerative component untreatable. This is in line with the TITAN Guidelines on the need for transparency in AI use in healthcare^[2]^.

Therapies based on nanotechnology have emerged as promising strategies to address the neurological complications of Gaucher disease type 3. Recent advancements have focused on lipid nanoparticles and polymeric carriers to ensure effective delivery of enzymes such as glucocerebrosidase across the BBB, facilitating the clearance of accumulated glucocerebroside. Intranasal administration has emerged as a promising and noninvasive route for this purpose^[3]^. Despite these advancements, the large-scale production of these vectors is challenging for a resource-limited country. Furthermore, additional clinical validation is required to establish their long-term safety and efficacy.

Nanotechnology-based therapeutic strategies in mice explore CNS targeting, minimally invasive delivery systems^[4]^. As reported by Ying *et al*, natural lipid-based formulations (~190 nm), having stabilized glucocerebrosidase, bypass the mannose receptors to breach the BBB. Treatment improved the clearance of accumulated glucosylceramide (*P* = 0.037) and helped in reducing inflammation^[4]^. Similarly, Janpipatkul *et al* engineered lentiviral carriers containing recombinant GBA1 that were internalized by different cell lines, including neurons, making BBB penetration possible^[5]^. Gehrlein *et al* achieved GCase escape across the BBB by fusion with the transferrin binding region, recovering lysosomal action^[6]^.

Despite the pioneering nature of the nanomedicine vector, some limitations need to be addressed. Although nanomedicine vectors have shown a beneficial effect in the mouse model, no clinical trials have yet been conducted to determine the efficacy in humans. A study by Sun *et al* demonstrated that earlier intervention at the time of symptoms onset might confer better outcomes, underscoring the need to determine the optimal treatment window^[4]^. Moreover, the exact mechanism of transport at the BBB remains unclear. Current studies emphasize that standardized cognitive metrics must be defined to assess the efficacy of neurotherapeutic trials and the clinical relevance of changes in cognitive performance^[7]^.

The possible use of nanomedicine for the treatment of neuropathic Gaucher disease type 3 is a groundbreaking way of addressing the gap in the treatment of its neurological manifestations, with the help of which the patients’ quality of life can be maintained. Conventional therapies such as enzyme replacement and substrate reduction cannot reach the brain because of their inability to cross the BBB. Innovative approaches based on nanotechnology, like lipid nanoparticles and viral vectors, have made it possible to deliver glucocerebrosidase to the central nervous system, thus improving disease management^[4,7]^. The letter aims to provoke more inquiries into the large-scale production techniques that would make the drug more widely available, especially in regions with limited resources, encourages conducting exhaustive clinical trials to prove long-lasting safety and efficacy in humans, and calls for the creation of cognitive metrics that would better evaluate therapeutic outcomes, thus moving the field toward more efficient and inclusive treatment options.

In conclusion, nanomedicine vectors provide a novel approach for neuropathic Gaucher disease, as current therapies are not BBB-permeable. Future researchers should aim for clinical trials to determine the efficacy, safety, and cost-effectiveness in humans. Parkinsonism has been identified as an early indicator of GBA1-associated pathology, as reduced glucocerebrosidase activity was observed in a Parkinsonian brain. These findings emphasize that future studies should focus on CNS-permeable strategies.

## Data Availability

Not applicable to this study.
